# The effect of *Glycyrrhiza glabra* L. on Primary Dysmenorrhea compared with Ibuprofen: A Randomized, Triple-Blind Controlled Trial

**DOI:** 10.22037/ijpr.2020.1100961

**Published:** 2019

**Authors:** Zahra Jafari, Majid Emtiazy, Farnaz Sohrabvand, Daryush Talei, Laleh Oveidzadeh, Mahboobeh Abrishamkar, Mahshid Meyssami, Mohammad Kamalinejad

**Affiliations:** a *Department of traditional medicine, Faculty of Iranian Traditional Medicine, Shahid Sadoughi University of medical sciences, Yazd, Iran. *; b *Department of Obstetrics-Gynecology and Infertility, Vali-e-Asr Hospital, Imam Khomeini Hospital Complex, Tehran University of Medical Sciences, Tehran, Iran. *; c *Department of Biostatistics, Medicinal Plant Research Center, Shahed University, Tehran, Iran. *; d *School of Pharmacy, Shahid Beheshti University of Medical Sciences, Tehran, Iran.*

**Keywords:** Primary dysmenorrhea, Glycyrrhiza glabra L., G. glabra, Traditional Persian Medicine, Herbal medicine, Ibuprofen

## Abstract

Primary dysmenorrhea is a common gynecological disorder in women of reproductive age. Despite the effective conventional treatments such as nonsteroidal anti-inflammatory drugs and oral contraceptives, researchers have always been looking for alternative drugs due to the adverse effects and limited efficacy of these medications. *Glycyrrhiza glabra L. (G. glabra)*, commonly known as Licorice, has been applied for a long time as a plant with multiple therapeutic potencies in Traditional Persian Medicine (TPM). This study was designed to evaluate the effect of the *G. glabra* on primary dysmenorrhea.

Sixty patients with moderate and severe dysmenorrhea were randomly divided into two groups; one group received 400 mg Ibuprofen tablets every 8 h and placebo syrup and the other received 5 cc of *G. glabra* syrup two times a day and placebo tablets. The patients took the drugs from the first day of menstruation to fifth for two consequent cycles. The primary pain intensity and its changes were evaluated in each group and compared between two groups.

The reduction of pain intensity was 5.85 (±3.11) in the *G. glabra* group compared with 6.92 (±1.87) in the Ibuprofen group (*p *< 0.001). No significant difference detected between the two groups (*p* = 0.151). No serious side effects were reported during the study.

This study suggests that we can use *G. glabra* to relieve pain in the patients with primary dysmenorrhea; although studies with a larger sample size may lead to more comprehensive perceptions about the efficacy of *G. glabra*.

## Introduction

Cyclic pain refers to pain with a definite relevance to the menstrual period (1). Dysmenorrhea is the most common cyclic pain (1) and is classified as primary or secondary. Primary dysmenorrhea refers to menstrual cramping without any evident pelvic pathology whereas secondary dysmenorrhea refers to painful menses associated with underlying pathology (2).

Primary dysmenorrhea affects as many as 60% of menstruating women (2). This complaint results from excessive or imbalanced amounts of prostanoids secreted from the endometrium during menstruation which leads to increased uterine contractions, increased basal tone, and increased active pressure (2). Uterine hyper contractility decreases the blood flow and increases peripheral nerve hypersensitivity thereby contributing to pain (3, 4).

In the late luteal phase, the reduction of progesterone level, triggers lytic enzymaticaction, resulting in a release of phospholipids such as arachidonic acid which can be converted to prostanoids and leukotrienes through Lipo-oxygenase and cyclo-oxygenase pathways (4).

It is thought that women suffering from primary dysmenorrhea have up-regulated COX enzyme activity and prostanoid synthase activity. This leads to the use of nonsteroidal anti-inflammatory drugs (NSAIDs), which act as COX enzyme inhibitors, for therapy (5).

Despite the useful effects of the conventional drugs such as anti-inflammatory, antipyretic and analgesic (6, 7), some studies have reported a failure rate of 20% to 25% for these treatments of primary dysmenorrhea (8). On the other hand, these drugs may be contraindicated or intolerable in some women with primary dysmenorrhea (9).

Considering the contraindications, side effects and limited efficacy of NSAIDs, investigation is needed to achieve safe and effective alternative methods. Medicinal plants have high acceptability and tolerability and can be an appropriate alternative for chemical drugs in some cases.

*G. glabra* is a plant with broad healing capabilities. It has a long history of application in herbal and traditional medicine and is still appreciated as a medicinal herb. The plant was known to Assyrians thousands of years ago, Egyptians used it for religious ceremonies and Chinese and Hindus were aware of its invigorating effects. The use of *G. glabra* as a part of a medical prescription dates back to (IV-III B.C.) by Theophrastus. Later in the first century A.D., Dioscorides, placed *G. glabra* among the 650 medicinal substances of vegetable origin listed in his *De Materia Medica* (10). *Glycyrrhiza *is composed of two Greek terms: *glúkos*, “sweet”, and *ríza*, “*root*”. It is known as *Sus *or *Shirin Bayaan *in Traditional Persian Medicine with the same meaning as *Glycyrrhiza*. It is recommended as an anti-inflammatory and analgesic remedy in *Avicenna’s Canon of Medicine*.* G. glabra* has been administrated as a plant to relieve the uterine pain by *Rhazes* in *Al-Hawi *and also by *Aghili Alavi Shirazi *in* Gharaabaadin E Kabir*. Furthermore, *G. glabra* is used in folk medicine of China to treat dysmenorrhea and effects of the herb active ingredients on the uterine tissue, studied in some trials (11, 12).

*G. glabra* is composed of active ingredients including flavonoids and triterpenoids with a variety of biological activity. Studies have indicated that *G. glabra* has antimicrobial activity against both Gram-positive and Gram-negative bacteria (13, 14). It acts as an anti-fungal (15), anti-tussive (16), and expectorant (17, 18), anti-allergic (19), anti-oxidant (20, 21), anti-carcinogenic (22), anti convulsant (23) and memory enhancer (24). It also possesses many endocrine properties such as Mineralocorticoid (25, 26) and Glucocorticoid activity (27), Estrogenic (28) and anti-androgenic (29) effects and even decreases serum prolactin (30). 

Other studies have demonstrated that certain concentrations of *G. glabra* root components can inhibit Cyclo-oxygenase and Lipo-oxygenase pathways and therefore reduce Leukotriene andProstaglandin synthesis (31). Analgesic, muscle relaxant and antispasmodic effects of *G. glabra* constituents have been also supported in some studies (11, 32-34). Given the mentioned effects, it seems that this plant will provide our goal in relieving pain.

In addition, this plant has long been used in patients with gastritis and peptic ulcer with significant therapeutic effects compared with conventional drugs (35, 36) so it seems to be a good choice in patients with primary dysmenorrhea who suffer from digestive problems and do not tolerate NSAIDs.

Considering the prevalence of dysmenorrhea, the importance of treatment in maintaining women’s health and performance, fewer complications of herbal remedies compared to synthetic drugs and the tendency of patients to use alternative therapies, this study aimed to examine the effects of a drug used in traditional Persian medicine, a product based on *G. glabra*, to relieve the menstrual pain.

## Experimental

*Trial design*

The study had two parallel interventional arms with a randomized, active controlled and triple-blind design. The study was conducted in Tehran, Iran from April to October 2016 at dormitories of Shahid Beheshti University.

*Ethical considerations*

The study protocol was approved by the Ethics Committee of Yazd Shahid Sadoughi University of Medical Sciences (Reference number: IR.SSU.REC.1394.121) and then registered in Iranian Registry of Clinical Trials** (**IRCT registration number: IRCT2015081323610N1). All the patients entered the trial after providing written informed consent.

*Material*

*G. glabra* roots were purchased from a medicinal herb market (Tehran, Iran). Taxonomic identification was confirmed by Mr. M. Kamalinejad. A voucher specimen (No. 8066) is stored at the herbarium of Shahid Beheshti University of Medical Scieinces, (Tehran, Iran).

*Preparation of the materials*

The *G. glabra* and placebo syrups and the placebo tablets were prepared in the Medicinal Herbs Laboratory of Shahid Beheshti School of Pharmacy (Tehran, Iran). 1000 g of *G. glabra* roots were washed and then placed in a beaker to be boiled with 4 liters of water for about 30 min; the mixturewas filtered after cooling down and condensed on bain-marie. Finally, 150 g of dry extractwas obtained and then the 15% syrup of *G. glabra* was made on the basis of USP formulation. The prescribed dose was determined based on PDR for herbal medicines (37). 

The placebo syrup was prepared using the pharmacopoeia simple syrup formula. Both syrups had the same color and were filled in identical plastic pets. We used *rosa damascena* essential oil 0.001% - the least amount with no pharmacologic effects- in both placebo and *G. glabra* syrups to cover the taste and smell. 400 mg Ibuprofen tablets of Arya co. was used as the drug for the active control arm of the study and the placebo tablets were also made in the same color, size, and shape.

*Standardization of G. glabra extract*

The *G. glabra* syrup was standardized based on total flavonoid content via spectrophotometry using aluminum chloride and rutin solution as a reagent and standard control, respectively. The total flavonoid content was 4.87 mg/mL of the syrup. Furthermore, the syrup was standardized according to the total phenol content of 7.05 mg/mL. The total phenol content was determined by the Folin-ciocalteu method, using gallic acid as the reagent (38-40). 

*Inclusion criteria*

The female students who lived in Al-Zahra dormitory, affiliated to Shahid Beheshti University of medical Sciences, Tehran between the ages of 18-25 years old with moderate to severe primary dysmenorrhea, were selected for the trial if they had regular menstrual cycles (every 21-35 days and bleeding 3 to 10 days for at least three recent cycles). The grade of dysmenorrhea was based on the Verbal Multidimensional Scoring System (41, 42) with the four following grades: Painless menstruation = 0, Painful menstruation with rare need for analgesics or limitation of the normal working ability = 1 (mild), Painful menstruation with influence on daily activity and use of analgesics for pain relief = 2 (moderate), Painful menstruation with significant limitation on daily activity, poor effect of analgesics, and systemic symptoms such as headache, tenderness, nausea, vomiting, and diarrhea = 3 (severe). The patients with scores of 2 or 3 (moderate to severe dysmenorrhea) were included.

*Exclusion criteria*

The patients who had pelvic pathology (including Endometriosis, Adenomyosis, Fibroids, Ovarian Cysts, Pelvic Inflammatory Disease, etc), known diseases (including Chronic Hepatitis, Cholestatic Liver Disease, Cirrhosis, Severe Renal Insufficiency, Diabetes Mellitus, Arrhythmias, Hypertension, Hypertonia and Hypokalemia) or any other disease with obligatory medical treatment during the study, were all excluded from the study. Further exclusion criteria consisted of existence of stressors (such as loss of a close relative and intense familial debate) 6 months prior to the enrollment, concomitant use of OCP, concurrent use of other products containing *G. glabra* and a history of allergy to *G. glabra* or Ibuprofen. Enrolled patients were also excluded if they took another painkiller that was not defined in the study, had drug intolerance or did not have a desire to continue the treatment for any reason.

*Intervention*

A total of 115 female students who had reported dysmenorrhea through announcements, were interviewed for inclusion criteria. Fifty two did not meet the inclusion criteria; some of them for irregular menses and others for experiencing grade 1 (mild) dysmenorrhea. Subsequently, eligible persons were provided a precise medical history considering items of the exclusion criteria; they also underwent ultrasonography or clinical examinations under the supervision of a gynecologist to diagnose probable pelvic pathologies. Three individuals were excluded in this step due to ovarian cysts or taking OCP. Finally, the patients entered the trial after confirmation of eligibility and providing written consent. Demographic information and history of dysmenorrhea including the pain severity in the last menstrual cycle were recorded before enrolling the study. The participants were randomly divided into two groups. One of the two groups, received 400 mg Ibuprofen tablets every 8 h and 5 cc of placebo syrup two times a day and the other group received 5 cc of *G. glabra* syrup (150 mg/mL) two times a day and placebo tablets every 8 h. The patients took the drug from the first day of menstruation to the fifth and for two consecutive cycles. They were asked to report the extreme pain intensity before taking the first dose and also the most pain relief after a maximum of two hours from receiving the last dose via the forms containing 10 cm visual analogue scale. Finally, 26 patients in the *G. glabra* group and 24 of the Ibuprofen group completed the intervention. Here participants, observers and analysts did not have any information about the type of the given drug within the groups. It should be noted that although the patients were permitted to use Acetaminophen if needed, they were advised to report the dose for ultimate comparison. They also completed the International Physical Activity Questionnaires (IPAQ) to be observed for the amount of physical activity.

*Efficacy assessment*

The enrolled patients were assessed for the severity of pain by a visual analogue scale. The visual analogue scale (VAS) is a widely used instrument for measuring pain (43); respondents had to specify their perception of pain intensity by indicating a position along a continuous 10 cm line before and after intervention.

*Adverse effects assessment*

The participants in both groups were asked to report any allergic or adverse effects and also any changes in menstrual cycles including the bleeding volume and the duration of menses.

*Randomization, blinding and allocation concealment*

A block-randomization list with the same but non-stratified blocks was used for assigning participants to the *G. glabra* or Ibuprofen groups. The researchers had no access to the randomization list until the statistical analysis had been completed. During this triple-blind trial, the participants, observers and analysts did not have any information about the treatment allocation within the groups. To achieve this goal we applied coded packages containing *G. glabra* syrup and placebo tablets on one hand and placebo syrup and Ibuprofen tablets on the other hand. The syrups and tablets had also the same appearance, color, smell and taste to support the allocation concealment and blinding design of the study.

*Sample size estimation and statistical analysis*

To detect a difference in 0.9 cm in pain score between two groups with α = 0.05, power = 80%, assuming a standard deviation (SD) of 1 cm and considering a probable drop-out rate, the sample size was calculated to be about 30 patients in each arm of the study.

Descriptive analyses were carried out to calculate the basic characteristics of the groups such as mean, variance, and standard deviation. Characteristics of the two groups were analyzed with independent *t*-test. Paired samples *t*-test used to compare the mean pain scores (OR severity of pain) before and after treatment within the groups; *p* values ≤ 0.05 were considered as statistically significant. An analysis of variance was conducted to compare the outcomes based on the physical activity. All of the statistical analyses were performed using the SPSS Version 23.

## Results

*The study flow*

**Figure 1:**
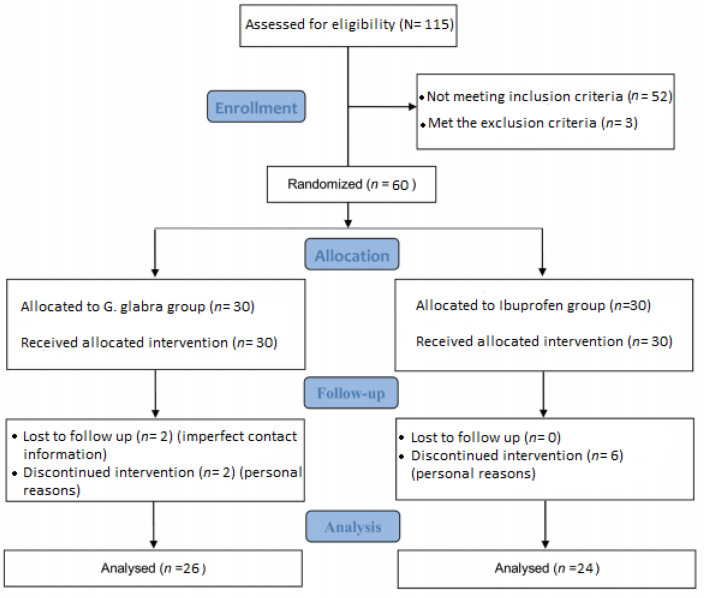
The CONSORT flowchart of trial

The process of recruitment, assessment and follow up of the participants began in April 2016 and ended in October 2016; total of 60 students who sampled from Tehran dormitories were randomly enrolled into two groups; 26 students in the *G. glabra* group and 24 in the Ibuprofen group completed the treatment process. The enrollment, treatment, and follow up procedure are presented in details in Figure 1.

*Participant characteristics*

The mean ± *SD* age of the participants was 22.60 ± 1.84 ranging from 19 to 25 years. Considering the baseline characteristics of the participants as age, BMI and menstrual pattern, there were no significant differences between the two groups as presented in Table 1.

*Efficacy assessment*

Table 2 exhibits the changes in mean values of the pain score in both intervention cycles and in general before and after the drug administration. The results showed a significant difference in the pain score before and after treatment in both groups of *G. glabra* (*p* < 0.001) and Ibuprofen (*p* < 0.001), but there was no significant difference in the pain relief between *G. glabra* and Ibuprofen groups (*p* = 0.151). The mean differences of the pain scores in before and after treatment, for each day of patients’ assessment and *p*-values are presented in Table 3; the pain score was also decreased significantly before and after intervention in both groups on daily evaluation. 

**Figure 2:**
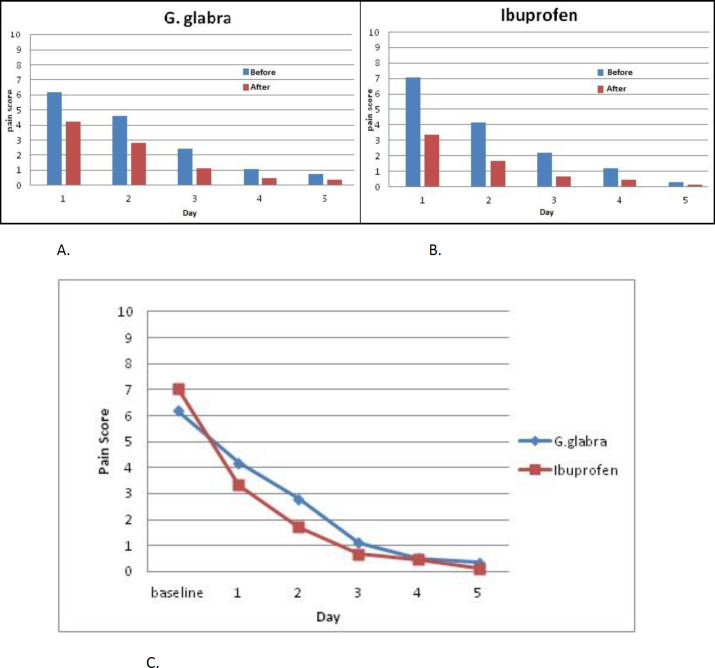
Trend of the mean values, before and after the drug administration in G. glabra (A) and Ibuprofen (B) groups in each day of the study. Decreasing trend of the pain score compared in intervention groups (C).

Figure 2 displays a visual comparison between the two groups by measuring the outcome mean values in each day of the study. Improvement in outcome measures suggests a therapeutic trend in both groups. No changes in the menstrual pattern were reported in the Ibuprofen group but 42.3 % of the participants in the *G. glabra* group reported a more diluted bleeding compared to their previous cycles. Side effects were reported in 6 participants (25%) in the Ibuprofen group, including heartburn (n = 5, 20.8%) and stomachache (n = 2, 8.3%). The *G. glabra* syrup was well tolerated with the participants.

On the other hand, we did not find a significant difference in pain relief between active, moderately active, and inactive participants (*p *= 0.248).

## Discussion

Phytochemical exploration of the *G. glabra* roots are in agreement with the results of the studies confirming the anti- inflammatory, anti- spasmodic, and analgesic effects of the components (31, 34). On the other hand, ethnopharmacological experiences approve its indication for menstrual pain (49- 51). To examine these effects we decided to conduct the present study on primary dysmenorrhea which is pathophysiologically related to up-regulated COX enzyme and prostanoid synthase activity (2, 5, 44).

Our study is a primitive investigation of the efficacy of *Glycyrrhiza glabra L*. (licorice) root extract on menstrual pain. To date there is no direct study about *G. glabra* and dysmenorrhea but conducted studies on the chemical components of the plant (31-34) have indicated effects in accordance with our study. In the present study administration of *G. glabra* root extract and Ibuprofen tablets both had similar positive effects on decreasing the pain severity of primary dysmenorrhea. The side effects of Ibuprofen were observed in a quarter of the participants and more participants discontinued the study, while there were no side effects in the *G. glabra* group and the syrup was well tolerated by the participants. 

Although a growing interest in herbal therapies, some challenges exist on their efficacy and safety; therefore there is an emerging need for evidence- based studies on their use (40). The results of this study also provided more evidence for the TPM. According to TPM *G. glabra* is introduced as an emmenagogue, analgesic, and anti-inflammatory (49- 51). Thus, uncomplicated positive effects of *G. glabra *are in line with TPM. Besides palliative effect and facilitating the menstrual blood flow in TPM perspective (49-51) could justify the effect of *G. glabra* on primary dysmenorrhea.

The results of the present study are also in accordance with previous studies for Ibuprofen as a nonsteroidal anti-inflammatory drug and a COX enzyme inhibitor. Regarding prior investigations the effectiveness of NSAIDS, as the first line medication for patients with primary dysmenorrhea, has been demonstrated in several studies (45-48) as well as indicated in our study. 

As for *G. glabra*, no study has directly evaluated the effect of the herb on primary dysmenorrhea. The previous investigations on *G. glabra* have explained the effect of isolated phytochemicals on separated animal tissues. For example Chandrasekaran *et al*. (31) reported that glabridin and isoliquiritigenin derived from *G. glabra* root were suppressors of prostaglandin biosynthesis through inhibition of Cyclo-oxygenase and Lipo-oxygenase pathways. Therefore, Ibuprofen and *G. glabra* were similar in suppressing prostaglandins, while our findings indicated that *G. glabra* could have the same therapeutic effects with less complication than NSAIDS. Moreover, Sato *et al*. (33) and Shi *et al*. (34) indicated the antispasmodic and uterine relaxant effects of isoliquiritigenin (a flavonoid isolated from the roots of *G. glabra*) respectively which are both are in line with our study results. Such plausible mechanisms for better understanding of the relaxant activity of isoliquiritigenin are voltage-dependent L-type Ca2+ channel blockade, the inhibition of NO synthase and the inhibition of PGs synthesis (34). Analgesic activity of isoliquiritigenin was also approved by Shi e*t al*. (34) which can be considered as an additional explanation for the palliative effects of the *G. glabra.*

In conclusion the results of this study showed that *G. glabra* syrup and Ibuprofen tablets had similar positive effects on reducing the severity of menstrual pain while *G. glabra* in addition to pain relief was safe and well tolerated so it could be a better choice than Ibuprofen in the treatment of primary dysmenorrhea. In other respect, according to the significant therapeutic effects of the *G. glabra* in patients with gastritis and peptic ulcer (35-36), it seems to be a good choice in the patients with primary dysmenorrhea who suffer from digestive problems and are not able to tolerate NSAIDS.

Studies with a larger sample size for a longer duration are required to attain a more comprehensive perception about the efficacy and safety of *G. glabra* in primary dysmenorrhea; Furthermore, the positive results of our study can be an incentive to design studies evaluating the effects of *G. glabra* on other symptoms associated with dysmenorrhea.

*Study limitations*

This study has some limitations that should be considered for interpretation and generalization of the findings. Some demographic and clinical characteristics of the patients, considered as inclusion limitations had to be taken into account for essential homogeneity of the study population in an interventional trial. 

The study is also limited by a lack of perception of the effects of *G. glabra* on the other symptoms associated with dysmenorrhea.

Moreover, if some of the participants did not take enough drug or at the right times it could biases the results to the null.

The short follow up duration was another limitation of the study and a longer duration may lead to achieve more details on the efficacy, safety, tolerability, and even adverse effects of the administrated drugs.

**Table 1.** Baseline participants’ characteristics in the two groups of G. glabra and Ibuprofen

**Parameters**		**G. glabra (** ***n = *** **26)** **Mean (±** ***SD*** **)**	**Ibuprofen (** ***n = *** **24)** **Mean (± ** ***SD*** **)**	***p*** **-value**
Age (years)		22.73 (1.91)	22.46 (1.79)	0.606
Body Mass Index (kg/m2)		22.22 (2.87)	21.99 (2.70)	0.775
Menarche Age (years)		13.27 (1.46)	13.08 (1.35)	0.643
Interval of cycles (days)		30.35 (3.23)	29.46 (2.96)	0.318
Duration of menses (days)		6.69 (1.29)	6.50 (1.06)	0.570
Pain score* (cm)	First cycle	6.31 (2.51)	7.33 (1.46)	0.087
	Second cycle	6.08 (3.26)	6.75 (3.21)	0.466

SD: Standard Deviation, p value is based on independent t-test

*The mean pain severity of the first day of menstruation based on VAS (Visual Analogue Scale)

**Table 2:** Comparison of the pain score before and after treatment within the groups

	**The mean pain score (± ** ***SD*** **)**					
	**G. glabra**			**Ibuprofen**		
	Before treatment	After treatment	*p*-value	Before treatment	After treatment	*p*-value
**The first cycle**	6.31 (2.51)	0.38 (0.94)	< 0.001	7.33 (1.46)	0.17 (0.56)	< 0.001
**The second cycle**	6.08 (3.26)	0.31 (0.84)	< 0.001	6.75 (3.21)	0.08 (0.28)	< 0.001
**Total assessment**	6.19 (2.52)	0.35 (0.88)	< 0.001	7.04 (1.70)	0.12 (0.30)	< 0.001

*SD*: Standard Deviation, The pain score is based on VAS (Visual Analogue Scale); *p*-value is based on paired *t*-test offering the difference in before vs. after VAS

**Table. 3:** Mean of the difference (±*SD*) of pain scores and within-group analysis of mean values for each time-spot

**Treatment Group**	**G. glabra**		**Ibuprofen**	
**Time**	Mean of difference (± *SD*)	*p*-value	Mean of difference (± *SD*)	*p*-value
**1st Day**	2 (1.48)	< 0.001	3.71 (1.86)	< 0.001
**2nd Day**	1.77 (1.31)	< 0.001	2.46 (1.61)	< 0.001
**3rd Day**	1.27 (1.22)	< 0.001	1.54 (1.84)	< 0.001
**4th Day**	0.54 (1.05)	0.013	0.75 (1.13)	0.004
**5th Day**	0.42 (0.98)	0.033	0.17 (0.38)	0.043

*p*-value is based on paired *t*-test offering the difference in before vs. after VAS in each day of the study
